# Examining the Gaze Behaviour and Decision‐Making of Field Hockey Players In Situ

**DOI:** 10.1002/ejsc.70062

**Published:** 2025-10-04

**Authors:** Bente M. van Dijk, Joris J. A. A. M. Hoeboer, Margot van Wermeskerken, Arie‐Willem de Leeuw, Sanne I. de Vries, David L. Mann

**Affiliations:** ^1^ Research Group Healthy Lifestyle in a Supporting Environment Centre of Expertise Health Innovation The Hague University of Applied Sciences The Hague The Netherlands; ^2^ Department of Education Utrecht University Utrecht The Netherlands; ^3^ Centre of Expertise Health Innovation The Hague University of Applied Sciences The Hague The Netherlands; ^4^ Department of Public Health and Primary Care Health Campus the Hague Leiden University Medical Centre The Hague The Netherlands; ^5^ Department of Human Movement Sciences Vrije Universiteit Amsterdam Amsterdam Movement Sciences and Institute Brain and Behavior Amsterdam (iBBA) Amsterdam The Netherlands

**Keywords:** decision‐making, field hockey, gaze behaviour, scan patterns

## Abstract

Gaze behaviour is associated with decision‐making in team sports. For instance, the final fixation of basketball players typically reflects the decision they make. However, it is not clear how athletes adapt in invasive team sports where they also control the ball using an implement (e.g., field hockey or lacrosse). In these sports, decision‐making might be related to information fixated earlier on rather than the final fixation. This study investigated the relationship between gaze behaviour and decision‐making in the dynamic sport of field hockey. We recorded the in situ gaze and decision‐making accuracy of 15 skilled youth field hockey players in specific 3 versus 3 small‐sided scenarios. Gaze behaviours were compared between players who made correct and incorrect decisions. Results indicated all players looked towards the ball in their final fixation before executing their decision, reflecting a critical difference from other invasive team sports such as basketball, where the ball carrier is in direct contact with the ball. Strikingly, it was the first fixation, rather than the last or second‐to‐last, in each scenario which was most associated with correct decisions. In particular, players who directed their first fixation towards the open space appeared most likely to make correct decisions in the scenarios we presented. The results emphasise the sport‐specific nature of gaze behaviour and raise doubts about the transferability of gaze behaviour between closely related sports. These findings contribute to our knowledge of gaze behaviour and the decision‐making processes of athletes in dynamic team sports.

## Highlights

- In *field hockey*, the first fixation was most associated with correct decisions.
- All players fixated on the ball before making a decision, highlighting the *sport‐specific nature* of gaze behaviour.
- Findings enhance the understanding of *gaze behaviour* and *decision‐making* processes of athletes during dynamic team sports.

## 1 Introduction

In the field of sport science, the analysis of gaze behaviour is increasingly popular as a means of examining where athletes concentrate their visual gaze and use visual information in their decision‐making process (Kredel et al. 2017). The focus on gaze behaviour aligns with the recognition that success in sport relies on optimal coordination between perception and action (Williams and Jackson 2019). This perception–action cycle (Gibson 1979) provides insight into how athletes continuously interact with and adapt to changes in their environment. By gathering relevant information from the environment through the perceptual system, athletes can make decisions to guide their actions. For instance, when a baseball player aims to catch a ball, they rely on picking up information about the ball's trajectory to anticipate and successfully catch the ball (Michaels and Oudejans 1992). Next, by virtue of their movement, the baseball player gathers new perceptual information to further guide their movement in a cyclical manner.

In dynamic (open) sports games (e.g., field hockey, soccer, basketball), the perception–action cycle is more complex compared to static (closed) sports games (e.g., gymnastics, athletics, golf). This complexity arises from the numerous variables that come into play during dynamic sports games and the changing position of the players in the environment (Inns et al. 2023). Static sports games require skills that are self‐paced and executed within stable environments that do not change during performance. On the contrary, open sports games entail sport skills that are externally paced and performed in dynamic environments that vary in terms of speed, direction, and levels of uncertainty (Farrell 1975; Poulton 1957).

Although many studies on sport performance have examined gaze behaviour and decision‐making in static sport scenarios such as a penalty in soccer (e.g., Savelsbergh et al. 2002), research in open sports games has received less attention (though see Roca et al. 2018; Kassem et al. 2022, 2024). Hence, much is still unknown about whether and how gaze behaviour is associated with decision‐making in more open and dynamic sports environments. To date, studies that have explored athletes' gaze behaviour in relation to decision‐making in dynamic situations often used simplified video‐based tests (Kredel et al. 2017) in which participants responded verbally or by pressing a button while wearing an eye‐tracking device without otherwise moving (e.g., Cañal‐Bruland et al. 2010; Mann et al. 2009; Ryu et al. 2013, 2015; Savelsbergh et al. 2002; Williams et al. 2006). Emerging evidence suggests that these tests may fall short of accurately reflecting the on‐field performance of athletes by providing an under‐representation or even misrepresentation of on‐field behaviour (Bruce et al. 2012; Dicks et al. 2010; Kassem et al. 2022, 2024; Mann et al. 2010; Van Maarseveen et al. 2015). Hence, in order to increase our insights into the role of gaze behaviour in dynamic situations, field studies are essential in which athletes perform tasks within a more representative experimental setting (Kredel et al. 2017).

van Maarseveen et al. (2018) were one of the first to study gaze behaviour in a representative dynamic situation, specifically, on a basketball court. They investigated where professional female basketball players looked when performing a pick‐and‐roll game in on‐court attacking scenarios, identifying key areas of interest (also referred to as AOIs) and basketball‐specific scan patterns (i.e., the order in which specific AOIs were looked at/fixated). Results demonstrated that most basketball players scanned for possible passing options and ultimately directed their gaze towards the option they chose at the end of the trial (i.e., shot, drive, roller, corner). In basketball, this is possible because the ball can be largely controlled by a skilled ball carrier without needing to direct their gaze towards the ball. In this sense, they have the attentional capacity to direct their gaze towards other areas of interest in the environment without having to direct gaze towards the ball.

The situation in basketball may be in contrast to a number of other dynamic team sports in which the player must control the ball using an implement. In field hockey and ice hockey, for instance, the player in possession of the ball needs to skillfully control the ball/puck while also scanning for possible opportunities to pass, shoot, or otherwise attack. In these situations, it is less clear how gaze is directed and how gaze informs subsequent actions. Conceivably, a player with good ball control may need less time to direct their gaze towards the ball, providing more time to allocate gaze towards other meaningful information in the environment. This, in turn, might lead to better decision‐making. Moreover, the need to look at the ball, particularly at the moment of hitting it, may require the player to make decisions earlier in time (i.e., before initiating the hit), making the pick‐up of early information critical. However, little, if anything, is known about the role of gaze behaviour in these open complex situations in which the player interacts with other players and simultaneously must control the ball using an implement. This knowledge will constitute a first step to developing targeted interventions that aim to improve field hockey players' gaze behaviour to ultimately improve their on‐field performance.

Therefore, the aim of this study was to investigate the relationship between gaze behaviour and decision‐making in the dynamic open‐sport game of field hockey. We measured the on‐field decision‐making of skilled youth field hockey players wearing a mobile eye‐tracker while placed in 3 versus 3 small‐sided attacking scenarios. Gaze behaviour was measured and compared for decisions that were judged to be correct or incorrect to identify potential gaze behaviours associated with success. A comparable study in basketball (van Maarseveen et al. 2018) found that the final fixation prior to ball release was predictive of the (correct) decision taken by the ball carrier. We expected differences between basketball and field hockey, given the requirement to look at the ball when hitting it in field hockey. Therefore, we sought to examine which fixations and scan patterns would be most critical in predicting success.

## 2 Materials and Methods

### 2.1 Participants

We recruited 15 highly skilled youth female field hockey players to take part in the study (M_age_ = 16.6 years, SD = 0.3; M_hockey experience_ = 11.0 years, SD = 0.2). Seven of the participants took part in under 18 competitions in the highest national league, and the other eight were highly talented female hockey players from the national under‐16 field hockey talent programme. The study was approved by the ethics committee of the Faculty of Behavioural and Movement Sciences of the Vrije Universiteit Amsterdam (file VCWE‐2021‐035) and all participants gave their written informed consent prior to the study.

### 2.2 Equipment

A Pupil Invisible mobile eye tracker (200 Hz; Pupil Labs, Berlin, Germany) was used to record the gaze behaviour of the participants. The glasses of the eye tracker were connected to a mobile recording unit, which was carried in an arm strap designed for holding a mobile telephone while running that enabled the participant to move freely. The Pupil Invisible (Pupil Labs; Tonsen et al. 2020) is a calibration‐free eye tracker. Before each measurement, the researcher checked whether the eye tracker was properly aligned by having the participant look at a fixed target and adjusted the gaze cursor manually if needed.

### 2.3 Procedure and Design

**FIGURE 1:**
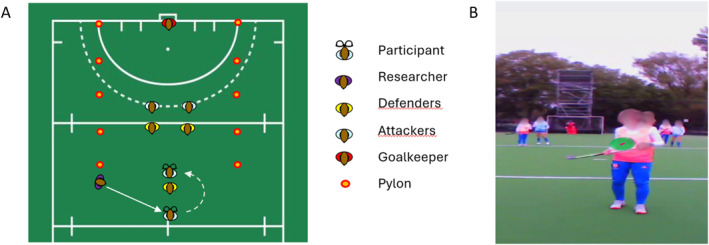
Demonstration of the starting positions for the attacking scenarios. Panel (A) shows a schematic demonstration of the participant (ball carrier) relative to other players. The pylons indicate the field in which the scenario was executed. Panel (B) shows a still image from the perspective of the eye tracker worn by a participant.

The test consisted of 3 versus 3 small‐sided attacking scenarios (i.e., three attackers vs. three defenders and a goalkeeper). The goalkeeper participated in the game to make the task as representative as possible. We designed four field hockey scenarios in collaboration with the head coach of the Dutch Under‐16 National Team to ensure that we examined realistic field hockey scenarios. Each scenario was designed in such a way that we were able to clearly observe whether the player made a correct or incorrect decision (see below). Games were played in good weather conditions (no rain and minimal cloud coverage) on a hockey field of 30 × 20 m. The participant, five players and goalkeeper started at specific locations (Figure 1) and played according to the official field hockey rules.

Before each trial, the three defenders and two attackers (but not the participant) were instructed by the researcher about which of the four scenarios they had to execute and on which side (left or right, involving one attacker and one defender) the action would be initiated towards the participant. Each trial started with a signal from the researcher followed by the participant receiving the ball. If the scenario was not executed as intended, for example, the ball rolled away or the players executed the wrong scenario, the trial was repeated.

The participant, wearing eye‐tracking glasses, completed 16 trials, four repetitions of each of the four scenarios presented in a random order and randomly alternating the scenarios between the right and left sides (see below). To simulate the defensive pressure experienced in the game, the participant had to dribble past the first defender. The defender was instructed to defend either the forehand or backhand of the participant. Once the participant passed the defender, the experimental scenario began. The participant had three options: 1. maintain possession and drive into open space; 2. pass to Attacker 1; or 3. pass to Attacker 2.

**FIGURE 2:**
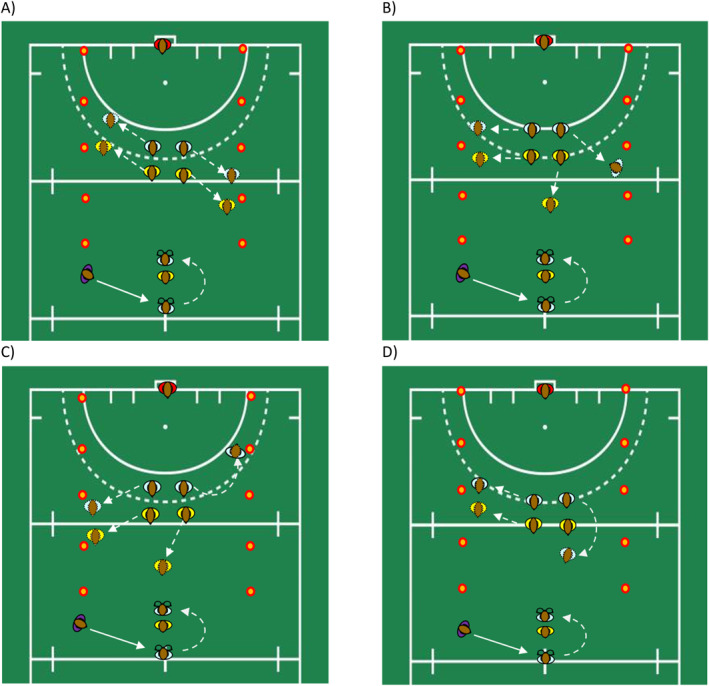
The four attacking scenarios used in the experiment. Panel (A) shows Scenario 1, where the correct response was to maintain possession and drive into open space. Panel (B) shows Scenario 2, where the correct response was to pass the ball to the unmarked attacker. Panel (C) shows Scenario 3, where the correct response was to pass the ball to the attacker who ran into space. Panel (D) shows Scenario 4, where the correct response was to pass the ball to the attacker who became free in front of their defender.

In Scenario 1 (see Figure 2, panel A), the two rear defenders marked the two attackers, who each moved to the sides of the field. The correct decision was made when the participant maintained possession of the ball and drove into the open space. In Scenario 2 (Figure 2, panel B), one of the two rear defenders stepped towards the participant, leaving the other attacker unmarked and able to receive a pass. A correct decision was made when the participant passed the ball to the unmarked attacker. In Scenario 3 (Figure 2, panel C), one rear defender and attacker moved to the side, whereas the second defender stayed in the middle and the attacker ran into the free space on the side. The correct decision was made when the participant passed the ball to the attacker who ran into the space. In Scenario 4 (Figure 2, panel D), one attacker and one rear defender moved to one side of the field, whereas the other attacker became free in front of their defender. A correct decision was made when the participant passed the ball to the attacker who became free in front of their defender.

In Scenarios 2, 3 and 4, the attacker and the defender who initiated the action towards the participant are always referred to as Attacker 1 and Defender 1, whereas the other attacker and defender are referred to as Attacker 2 and Defender 2. In Scenario 1, the left attacker and defender in the starting position, as perceived by the participant, are Attacker 1 and Defender 1, respectively. The right attacker and defender in the starting position are Attacker 2 and Defender 2, respectively.

### 2.4 Data Analysis

#### 2.4.1 Eye‐Tracking Measures

**FIGURE 3:**
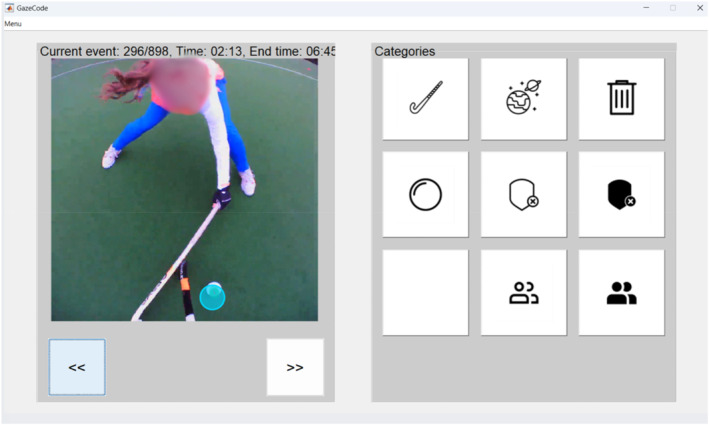
Screenshot of the Gazecode MATLAB interface. The left panel shows a frame from the scene camera of the Pupil Invisible eye tracker. The right panel shows the seven possible Areas of Interest (The trash bin was used when a fixation could not be utilised) to be manually coded for each fixation.

Raw data from the eye tracker were exported from Pupil Cloud. The data and scene videos were then imported into Gazecode in MATLAB (Benjamins et al. 2018), a programming package that allows each fixation to be mapped onto an Area of Interest (AOI) (Figure 3). In line with previous research, fixations were defined as having a maximum dispersion of 3° of visual angle and a minimal duration of 100 ms (van Maarseveen et al. 2016; Vickers 1996; Williams et al. 1999). To map each fixation within designated Areas of Interest using Gazecode, seven AOIs were identified for each scenario, based on the expertise of the coach of the Dutch Under‐16 National Team: 1. the ball; 2. Defender 1; 3. Defender 2; 4. Attacker 1; 5. Attacker 2; 6. space; and 7. the hockey stick (of Attacker 1). Subsequently, using Gazecode, each fixation was mapped onto one of the AOIs manually by the first and second author, by watching each individual trial for each individual participant. For each video, trial start was defined as the moment at which the participant looked up after having passed the first defender and trial end was defined as the moment at which the participant released the ball (for a drive or pass). After this mapping process, Gazecode provides an output file that entails a time series with all fixation data and their corresponding AOIs, which is then further used for analysis.

To gain insights into the gaze behaviour, we conducted several analyses. To ensure the quality of the coded AOIs, the interobserver reliability was determined for all 803 fixations for agreement between the first and second authors; the level of agreement was high (Kappa = 0.95, *p <* 0.001).

We were interested in the scan patterns the players displayed and therefore identified the frequency of transitions from one AOI to another, that is., two‐fixation scan patterns, rather than a complete scan pattern. Markov matrices were created to gain insights into the two‐fixation scan patterns of the players. Re‐fixations at the same location were excluded from the analysis, as the focus was solely on transitions between different AOIs.

#### 2.4.2 Statistical Analysis

To investigate whether gaze behaviour was different for correct and incorrect decisions, we analysed the frequency distribution on AOIs for the first fixation (i.e., the first fixation after looking up from the ball) and the second‐to‐last fixation (i.e., the final fixation just before gaze was directed at the ball) for correct and incorrect decisions and for each scenario. The following procedure was used: First, we determined the number of fixations directed at each AOI (i.e., Attacker 1, Attacker 2, Defender 1, Defender 2, ball and space) for correct and incorrect decisions. Second, to investigate whether the distribution of fixations was different for correct and incorrect decisions for a given AOI, a 2 × 2 contingency table was created that included the fixation frequency on this AOI for correct and incorrect decisions and the sum of fixations on all other AOIS for correct and incorrect decisions. *Fisher's exact tests* were used to assess significant differences in frequency distribution, with a significance level set at 0.05. Since multiple tests (5 or 6) were performed on the same data (i.e., for each scenario and each AOI), a Holm–Bonferroni correction was applied.

Finally, we used Cramer's V to determine the effect sizes and corresponding 95% confidence intervals. We considered the effect sizes to be small (Cramer's V < 0.2), medium (0.2 < Cramer's V < 0.6) or large (Cramer's V > 0.6). The 95% confidence intervals were obtained by using bootstrap resampling.

## 3 Results

### 3.1 Distribution of the AOIs

In 241 trials, a total of 803 fixations were recorded and categorised into six different areas of interest (AOIs). Therefore, the average number of fixations per trial was 3.3 fixations. During data collection, we inadvertently recorded 17 scenarios for one participant instead of the intended 16. As a result, one extra scenario was conducted, leading to a total of 241 trials instead of the planned 240 trials.

The ball received the highest number of fixations, with 317 out of 803 fixations (39%). The open space was the second most fixated area, with 231 out of 803 fixations (29%), followed by Defender 1 (84 out of 803, 10%), Attacker 1 (59 out of 803, 7%), Defender 2 (57 out of 803, 7%) and Attacker 2 (55 out of 803, 7%). None of the players directed their gaze towards the hockey stick during the 241 trials. In the remainder of the analysis, the hockey stick is disregarded as an AOI.

### 3.2 Last and Second‐to‐Last Fixation

All field hockey players fixated the ball as their last fixation before playing the ball. Considering that the players fixated on the ball during their last fixation, rather than one of the options they chose, we proceeded to determine whether there was a direct relationship between the players' gaze in the second‐to‐last fixation and their decision (see Table 1). When comparing the second‐to‐last fixation location for the correct and incorrect decisions, no significant differences were found for Scenario 1, Scenario 2 or Scenario 3 after applying the Holm–Bonferroni correction for multiple comparisons. However, the Fisher's exact test did reveal a significant difference for Scenario 4, with more incorrect decisions being made when the second‐to‐last fixation was directed at Defender 2 (*p <* 0.01; Cramer's V = 0.35, 95% CI: [0.20, 0.50]). The decision in Scenario 4 was more often correct when the second‐to‐last fixation was on ‘Attacker 1’, who was the player who should receive the ball (*p <* 0.01; Cramer's V = 0.59, 95% CI: [0.43, 0.75]).

**TABLE 1:** Frequency distribution for the location of the second‐to‐last fixation in each of the four scenarios.

Scenario 1					
Decision	Attacker 1	Attacker 2	Defender 1	Defender 2	Space
Correct	1	4	4	1	19
Incorrect	2	3	5	3	10
*p*‐value	0.58	1	0.49	0.31	0.16
Cramer's V (95% CI)	0.15 (0.01–0.39)	0.09 (0.01–0.27)	0.14 (0.00–0.41)	0.18 (0.01–0.35)	0.23 (0.02–0.58)

Scenario 2					
Decision	Attacker 1	Attacker 2	Defender 1	Defender 2	Space
Correct	5	0	13	3	17
Incorrect	1	3	3	4	9
*p*‐value	0.65	0.04	0.22	0.22	1
Cramer's V (95% CI)	0.16 (0.01–0.31)	0.32 (0.16–0.55)	0.24 (0.02–0.42)	0.18 (0.02–0.47)	0.11 (0.01–0.30)

Scenario 3					
Decision	Attacker 1	Attacker 2	Defender 1	Defender 2	Space
Correct	6	2	14	1	16
Incorrect	1	4	5	4	6
*p*‐value	0.40	0.17	0.56	0.04	0.57
Cramer's V (95% CI)	0.17 (0.03–0.34)	0.23 (0.03–0.44)	0.13 (0.01–0.32)	0.29 (0.03–0.51)	0.12 (0.00–0.34)

Scenario 1					
Decision	Attacker 1	Attacker 2	Defender 1	Defender 2	Space
Correct	13	2	5	0	6
Incorrect	0	6	4	8	14
*p*‐value	< 0.01^a^	0.28	0.72	< 0.01^a^	0.16
Cramer's V (95% CI)	0.59 (0.43–0.75)	0.18 (0.02–0.37)	0.12 (0.01–0.29)	0.35 (0.20–0.50)	0.22 (0.03–0.43)

^a^ Significance after Holm–Bonferroni correction.

### 3.3 First Fixation

It is plausible that players in Scenarios 1, 2 and 3 did not direct their gaze towards the expected AOIs during the second‐to‐last fixation, potentially due to prior attention given to those AOIs. Therefore, Table 2 shows the difference in the players' first fixation locations before making a correct or incorrect decision for each of the four scenarios.

**TABLE 2:** Frequency distribution tables for the location of the first fixation in each of the four scenarios.

Scenario 1					
Decision	Attacker 1	Attacker 2	Defender 1	Defender 2	Space
Correct	2	0	1	1	25
Incorrect	2	4	3	6	8
*p*‐value	1	0.03	0.31	0.04	< 0.01^a^
Cramer's V (95% CI)	0.12 (0.01–0.31)	0.34 (0.18–0.50)	0.18 (0.01–0.37)	0.34 (0.07–0.48)	0.54 (0.28–0.77)

Scenario 2					
Decision	Attacker 1	Attacker 2	Defender 1	Defender 2	Space
Correct	5	2	5	0	26
Incorrect	1	1	1	4	13
*p*‐value	0.65	1	0.65	0.01	1
Cramer's V (95% CI)	0.14 (0.01–0.32)	0.12 (0.01–0.29)	0.16 (0.00–0.32)	0.37 (0.20–0.55)	0.09 (0.01–0.29)

Scenario 3						
Decision	Attacker 1	Attacker 2	Ball	Defender 1	Defender 2	Space
Correct	2	0	2	5	2	29
Incorrect	3	2	0	3	3	9
*p*‐value	0.32	0.11	0.55	1	0.32	0.05
Cramer's V (95% CI)	0.18 (0.00–0.38)	0.27 (0.16–0.47)	0.14 (0.08–0.23)	0.09 (0.00–0.23)	0.18 (0.02–0.42)	0.29 (0.07–0.54)

Scenario 4					
Decision	Attacker 1	Attacker 2	Defender 1	Defender 2	Space
Correct	10	0	2	0	14
Incorrect	3	8	2	4	15
*p*‐value	0.01^a^	< 0.01^a^	1	0.12	0.79
Cramer's V (95% CI)	0.35 (0.10–0.57)	0.35 (0.23–0.49)	0.12 (0.01–0.27)	0.23 (0.12–0.38)	0.12 (0.01–0.36)

^a^ Significance after Holm–Bonferroni correction.

The results indicate a difference in the first fixation when players made a correct decision compared to an incorrect decision in two of the four scenarios. In Scenario 1, in which the correct decision was to move into space, players who made a correct decision more often fixated the AOI ‘space’ during their first fixation than players who made an incorrect decision (*p <* 0.01; Cramer's V = 0.54, 95% CI: [0.28, 0.77]). In Scenario 2, the decision was more often incorrect if the first fixation was towards Defender 2, that is, away from what was ultimately the correct direction, although this finding did not reach significance after applying the Holm–Bonferroni correction for multiple comparisons (*p* = 0.01; Cramer's V = 0.37, 95% CI: [0.20, 0.55]). In Scenario 3, where the correct decision was to pass to Attacker 1 who moved into free space, players who first looked at the free space were almost three times more likely to make the correct decision. However, the effect just fell short of significance after applying the Holm–Bonferroni correction for multiple comparisons (*p* = 0.05; Cramer's V = 0.29, 95% CI: [0.07,0.54]). Finally in Scenario 4, in which the correct decision was to pass to Attacker 1 in front of their defender, players fixated more often on Attacker 1 when making a correct decision (*p* = 0.01; Cramer's V = 0.35, 95% CI: [0.10, 0.57]), and more often on Attacker 2–who was not involved in executing an action towards the player–when making an incorrect decision (*p* < 0.01; Cramer's V = 0.35, 95% CI: [0.23, 0.49]).

### 3.4 Scan Patterns Across Different Scenarios

Figure 4 shows the scan patterns for each scenario. The circles represent the six fixation locations; the size of the circle represents the percentage of fixations towards that location, also numerically displayed under the name of the fixation location. The arrows represent the fixation transitions; the thickness of the arrow represents the probability of the fixation transition, also displayed numerically beside the arrows. A green (correct) or red (incorrect) arrow indicates that the probability of this transition was 25% higher than the probability of the same transition when the alternative decision was made in that scenario. For example, in Scenario 1, the probability of fixating the ball after having fixated Defender 2 was 0.60 for those who made the incorrect decision, whereas it was 0.29 for those who made the correct decision. The sum of the weights of the outgoing arrows from each AOI equals 1. Table 3 shows the sum of the weights of the incoming arrows. The incoming arrows can have weights smaller or larger than one, indicating how important the AOI was in that scenario.

FIGURE 4Scan patterns in gaze behaviour between correct and incorrect decisions for Scenarios 1–4. The circles represent the six fixation locations; the size of the circle represents the percentage of fixations towards that location, also numerically displayed under the name of the fixation location. The arrows represent the fixation transitions; the thickness of the arrow represents the probability of the fixation transition, also displayed numerically beside the arrows. A green (correct) or red (incorrect) arrow indicates that the probability of this transition was 25% higher than the probability of the same transition when the alternative decision was made in that scenario.

**Figure:**
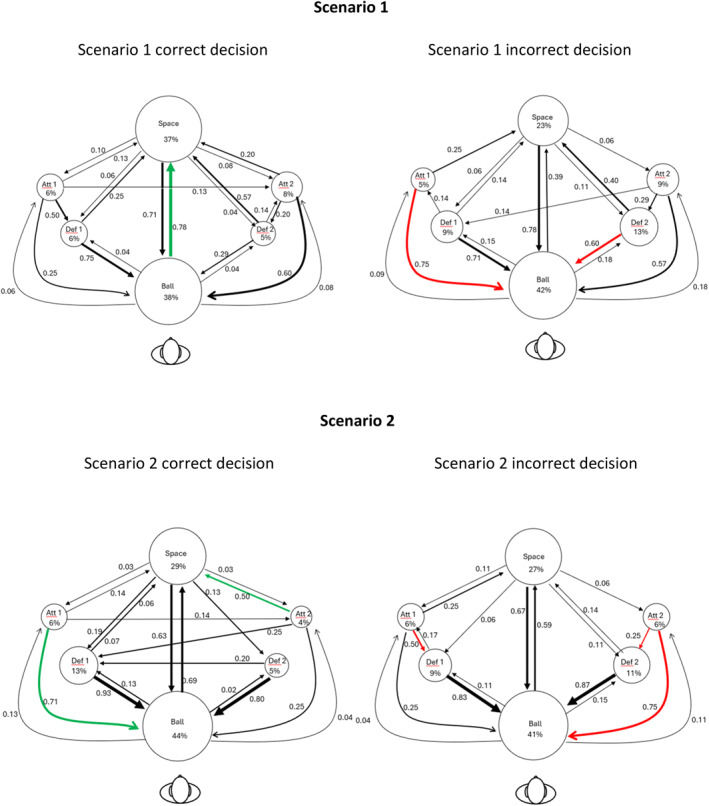

**Figure:**
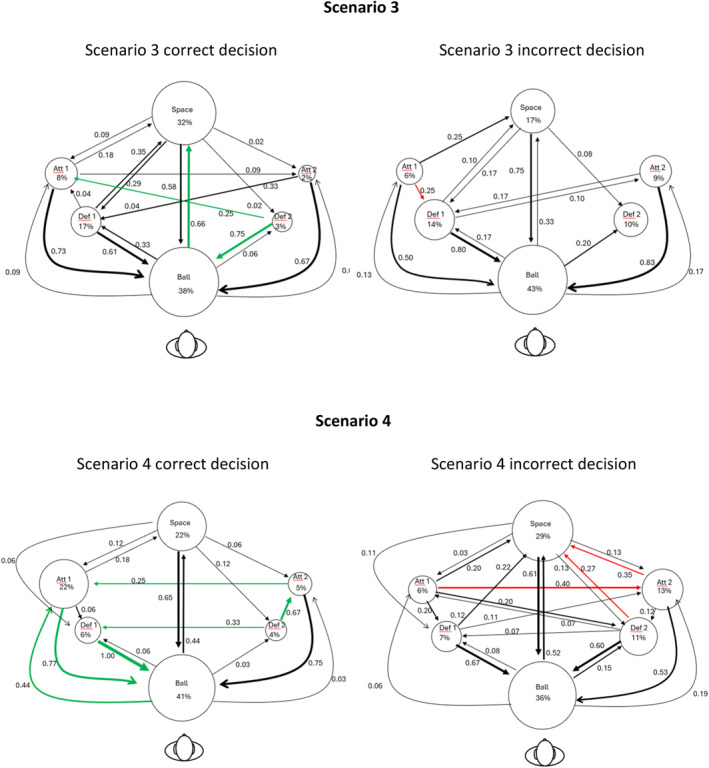

**TABLE 3:** The sum of the weight of incoming arrows in four scenarios.

Scenario 1						
Decision	Attacker 1	Attacker 2	Ball	Defender 1	Defender 2	Space
Correct	0.16	0.43	2.59	0.60	0.28	1.96
Incorrect	0.23	0.24	3.41	0.35	0.58	1.19

Scenario 2						
Decision	Attacker 1	Attacker 2	Ball	Defender 1	Defender 2	Space
Correct	0.16	0.22	3.32	0.76	0.15	1.40
Incorrect	0.32	0.18	3.36	0.67	0.51	0.99

Scenario 3						
Decision	Attacker 1	Attacker 2	Ball	Defender 1	Defender 2	Space
Correct	0.48	0.13	3.33	0.96	0.08	1.19
Incorrect	0.13	0.27	2.88	0.75	0.28	0.68

Scenario 4						
Decision	Attacker 1	Attacker 2	Ball	Defender 1	Defender 2	Space
Correct	0.81	0.76	3.16	0.51	0.15	0.61
Incorrect	0.16	0.83	2.40	0.46	0.60	1.56

*Note:* The sum of the weights of the outgoing arrows from each AOI equals 1. The incoming arrows can have weights smaller or larger than one, indicating how important the AOI was in that scenario.

In Scenario 1, in which the correct decision was for the ball carrier to move into space, the fixated space appears to be an important factor for making a correct decision. The probability of transitioning gaze from the ball to open space was 0.78 for a correct decision, compared to 0.39 for an incorrect decision. In addition, in the sum of the weighted arrows, we observe that for correct decisions, there was a stronger emphasis on the AOI space compared to an incorrect decision. This means that when making a correct decision, more arrows with higher weight were directed towards ‘space’ than for incorrect decisions. In contrast, for incorrect decisions, there were higher probabilities of transitioning gaze from Attacker 1 to the ball and from Defender 2 to the ball.

In Scenario 2, where the correct decision was to pass to Attacker 1 who moved into open space, participants who made a correct decision were more likely to shift their fixation from Attacker 1 to the ball, whereas those who made an incorrect decision were more likely to direct their gaze from Attacker 2 to the ball. When summing the arrows pointing towards the space, we see also a difference between the correct and incorrect decisions, with an increased probability of transitioning gaze to the space for the correct decisions when compared to the incorrect decisions (the total weight of arrows = 1.40 vs. 0.99).

In Scenarios 3 and 4, multiple transitions were associated with making a correct decision. In Scenario 3, in which the correct decision was to pass to Attacker 1 who moved into free space, the transitions for a correct decision were quite varied, making it challenging to identify a clear pattern. For correct decisions, more transitions were made from the ball to the open space and marginally more from Attacker 1 to the ball. Moreover, the sum of the weighted arrows indicates that the transitions of gaze towards the AOI space were associated with success.

In Scenario 4, in which the correct decision was to pass to Attacker 1 in front of their defender, a clear difference in scan patterns was evident. When making an incorrect decision, many transitions occurred on the right side of the figure between Attacker 2 and space and between Defender 2 and space. In contrast, for a correct decision, many transitions moved between Attacker 1 and the ball.

Furthermore, there was a difference in the number of fixations towards the different locations (i.e., the sizes of the circles) across the correct and incorrect decisions. During an incorrect decision, players fixated more often on Defender 1 and Defender 2, who were not actively engaged in this scenario. The sum of the weighted arrows also highlights this pattern. For a correct decision, Attacker 1 had a weight of 0.81, whereas for an incorrect decision, this weight dropped to 0.16. In addition, the role of space became less important when making a correct decision.

## 4 Discussion

The aim of this study was to investigate the relationship between gaze behaviour and decision‐making in the dynamic open‐sport game of field hockey. Fifteen youth hockey players each took part in four dynamic 3 versus 3 attacking scenarios that were designed to be representative of situations that are commonly encountered in field hockey. The decisions of the participants were assessed relative to the decision chosen to be correct for each scenario, and gaze behaviour was recorded during play using a mobile eye tracker. Subsequently, we examined the first fixation, the second‐to‐last fixation, the final fixation and the overall scan patterns of their gaze behaviour. Results revealed significant associations between gaze behaviour and decision‐making success. In contrast to previous studies which found the final fixation before action execution to be associated with success (e.g., van Maarseveen et al. 2018), we instead found that it was the first fixation made in a given scenario that was most distinctive for correct and incorrect decisions. The results suggest that gaze behaviour might be associated with decision‐making in a dynamic team sport such as field hockey.

Several studies have shown that the final fixation before ball release is associated with success in sport tasks. In aiming tasks, such as a basketball free throw or a golf putt, the duration of the final fixation prior to ball release has been shown to be predictive of the success of the movement outcome (Harle and Vickers 2001; Vine et al. 2011). The final fixation has also shown to be critical in decision‐making tasks in sports. van Maarseveen et al. (2018) showed that basketball players in on‐court scenarios fixated on the option they chose at the end of the trial. For example, if a player decided to pass to a particular teammate, they directed their gaze towards that teammate just before making the pass. The results in our hockey decision‐making task were quite different. In field hockey, every player looked at the ball immediately before making their final decision. This difference is likely due to the fact that the ball carrier in field hockey is not in direct contact with the ball. Instead, they are using a stick to control the ball and likely need to direct their gaze at the ball in order to hit it in the direction they want to. The requirement to look at the ball probably means that players need to predict earlier in the process where a ball receiver will be in the near future in order to receive the ball. In basketball, however, the direct contact with the ball likely makes it easier for basketball players to look up while still maintaining control of the ball. Consequently, the second‐to‐last fixation in field hockey may correspond to the final fixation in basketball, albeit with a great time delay between the fixation and ball release.

Surprisingly in the majority of the hockey scenarios, the second‐to‐last fixation was not predictive of the correctness of the decision. In just one of the four scenarios was there a significant association between where participants looked in the second‐to‐last fixation and the success of the decision (correct or incorrect). Specifically, in Scenario 4, participants who made correct decisions were more likely to direct their gaze towards the AIO related to the correct option in that scenario (i.e., Attacker 1) during the second‐to‐last fixation than participants that made incorrect decisions. In the other three scenarios, no association was found between decision‐making and the direction of the second‐to‐last fixation.

Strikingly, it was actually the first fixation that was most predictive of successful decision‐making rather than the second‐to‐last fixation or last fixation. For each of the four scenarios, there was almost always an association between the direction of the first fixation and the correctness of the decision. In Scenario 1, the correct option for the participant was to move into space, and indeed those who did so were more likely to direct their first fixation towards space. In Scenario 2, those who made an incorrect decision were marginally more likely to look towards Defender 2 with their first fixation (*p =* 0.01, just short of statistical significance after the Holm–Bonferroni correction), whereas those who made a correct decision were more likely to direct their gaze towards space or other players. In Scenario 3, it shows that those who correctly passed to Attacker 1 moving into space were more likely to direct their first fixation towards space (though the difference was not significant, *p* = 0.05). And finally in Scenario 4, participants who correctly passed to Attacker 1 in front of their defender were significantly more likely to direct their first fixation towards Attacker 1 and less likely to direct it towards Attacker 2. In each case, the location of the first fixation seems to be strongly associated with the correctness of the decision. Early fixations on the wrong location seem to be associated with more frequent incorrect decisions. For example, in Scenario 2, initial fixations towards Defender 2 were associated with incorrect decisions. It is important to point out though that there is not necessarily a one‐to‐one relationship between where participants precisely looked and the decision that they made. In particular, the (correct) decision in Scenario 4 to pass to Attacker 1 who moved into space was associated with an increased likelihood of indeed directing the first fixation being directed towards Attacker 1. However, the (correct) decision in Scenario 2 to pass to Attacker 1 moving into space was associated with the first fixation being directed towards the space rather than the attacker. In both cases, the first fixation was associated with the correct decision, but the direction of gaze did not exactly specify the exact decision, or the person towards which the ball might be passed.

The tendency to direct gaze towards open space was often associated with success in our hockey decision‐making task. First, the highest percentage of all first fixations in our study was directed towards the open space. In Scenarios 1, 2 and 3, a higher percentage of first fixations on the space was observed for correct decisions compared to incorrect decisions. In Scenario 1, a significant difference was found between making a correct versus an incorrect decision when the space was the first fixation. In Scenario 2 and 3, the differences were not statistically significant. However, in Scenario 2, participants were twice as likely to first look towards the space when making a correct decision. In Scenario 3, the difference was substantial with participants being three times more likely to first look at open space when making a correct decision, although this finding did not reach significance. In addition, the sum of the weights of fixation transitions in Table 3 indicates that space is a crucial AOI for making a correct decision. For instance, in Scenario 3, the total sum of transitions towards space was 1.19 for a correct decision and 0.68 for an incorrect decision. This means that transitions were more frequent from one AOI to the space during a correct decision. The tendency to direct gaze towards open space seems to be an important factor in decision‐making success in open‐team sports (see also Mann et al. 2009). Studies that fail to code gaze towards open space (e.g., when using only pre‐defined areas of interest) may miss out on this potentially important information that seems to be associated with success in decision‐making.

It might be tempting to conclude that it is the direction of the first fixation that leads to the decision made by participants. To do so would imply causation: that it is the direction of the first fixation that *causes* a particular decision to be made and therefore that gaze predicts the decision outcome. This conclusion can be expected based on, for instance, the *take‐the‐first heuristic* which suggests that decision‐making in complex scenarios can be simplified by choosing to take the first decision that presents itself to a skilled player (Raab and Johnson 2007). Although we believe that the first fixation does indeed seem to be influential in decision‐making, the location of that fixation is not entirely predictive of the decision being made. First, participants in our study did not always choose the first option that they looked at. There was indeed an association, but this was not always the case. Second, there was not necessarily a one‐to‐one relationship between where participants precisely looked and the decision that they made. In particular, the (correct) decision in Scenario 4 to pass to Attacker 1, who moved into space, was associated with an increased likelihood of indeed directing the first fixation towards Attacker 1. However, the (correct) decision in Scenario 2 to pass to Attacker 1 moving into space was associated with the first fixation being directed towards the space rather than the attacker. In both cases, the first fixation was associated with the correct decision, but the direction of gaze did not exactly specify the exact decision, or the person towards which the ball might be passed. Therefore, there is some ambiguity in the direction of gaze and the decision taken. We think it is most parsimonious to conclude that the first fixation was very influential but not necessarily deterministic or final in our hockey decision‐making task.

The second‐to‐last fixation was also somewhat associated with the success of the decision, but not so much as the first fixation. Participants in Scenario 4 were more likely to correctly choose to pass to Attacker 1 if they directed their second‐to‐last gaze towards Attacker 1, and less towards Defender 2. However, this was the only scenario with a significant association for the second‐to‐last fixation. In contrast, two of the four scenarios had significant associations for the first fixation, whereas the other two scenarios had marginally significant associations. Moreover, the effect sizes for Scenarios 1, 2 and 3 (as quantified by the Cramer's V) were larger for the first fixation than for the second‐to‐last fixation. Although this was not the case for Scenario 4, there were still two significant associations with moderate values for Cramer's V (> 0.35) for both the first fixation and second‐to‐last fixation. Although there are significant associations between decision‐making success and the location of both the first and second‐to‐last fixation, it is the first fixation that appears most predictive of success.

The association between the direction of the first fixation and success in our hockey decision‐making task raises questions about why some players succeed in this task while others do not. For instance, why do some players look in the ‘correct’ direction with their first fixation? Are they able to pick up on some form of ‘scene gist’ (Oliva and Torralba 2006) or ‘pattern recognition’ (Gorman et al. 2012) that leads them to the best possible solution without a need for search? Might it be that they are better able to pick up crucial task‐specific information using their peripheral vision (e.g., see Ryu et al. 2013; Vater et al. 2022), or might others get distracted by the movement of other players in the field rather than looking towards space? These questions might be best answered in future studies by manipulating information available in the environment to see what draws attention and what does not.

This will help to investigate when and how the decision‐making of players can be improved by altering their first direction of gaze in decision‐making scenarios, for example, by in‐ear coaching or real‐time vibrotactile feedback. If we are able to understand the initial gaze directions most associated with success, then it might be possible to educate and coach players to alter their gaze (explicitly or otherwise).

It is interesting to compare the scan patterns we have found with those found by van Maarseveen et al. (2018). Our Scenario 1 was based on the man‐marking scenario that van Maarseveen et al. investigated in which each teammate was closely marked by a defender.

In van Maarseveen et al.’s study, the scan patterns revealed differences between correct and incorrect decisions. Incorrect decisions were characterised by a higher number of fixation transitions from more remote fixation locations (i.e., the basket and the corner defender), whereas correct decisions were more likely when transition occurred between players in more central fixation locations. Both studies seem to highlight the crucial role of open space.

However, due to the different body postures involved, it remains difficult to make a direct comparison of the scan patterns. For example, in van Maarseveen et al.’s study, only 0.8% of fixations during correct decisions were directed towards the ball, whereas in the field hockey scan patterns, at least 38% of fixations were directed towards the ball. This finding suggests that gaze behaviour and the perception–action behaviour cannot easily be transferred from one sport to another.

In summary, the scan patterns and the first fixations of field hockey players revealed that there is a distinction in gaze behaviour between correct and incorrect decisions made by the players. Moreover, the results highlight that gaze behaviour is sport‐specific, influenced by the unique constraints of each sport. Moore and Müller (2014) suggested that gaze behaviour could be transferred between closely related sports. Our study suggests that such transfer might only be feasible if the sports share comparable postures and ball control.

It was the first fixation in the open space that was most decisive of whether field hockey players made the correct decision or not. Peripheral vision may play a role in this context. According to the study conducted by Ryu et al. (2013), elite male basketball players do not only use central vision but also their peripheral vision. In their study, the involvement of central and peripheral vision in a gaze‐contingent paradigm was investigated during a video‐simulated decision‐making task. Both skilled and less‐skilled basketball players were shown video clips of basketball scenarios under different visual conditions: full vision, central vision only or peripheral vision only. After each clip, the participants had to indicate whether it was better for the ball carrier to pass to a teammate or drive to the basket. The results revealed that skilled players outperformed the less‐skilled players regardless of whether they had full vision or were limited to either central or peripheral vision alone. This indicates that skilled athletes can use both their central and peripheral vision effectively in decision‐making. Peripheral vision may provide an explanation for why the hockey players in our study did not fixate on their teammates in Scenario 1 before making a correct decision. By scanning the surrounding space, players may have been able to perceive their teammates moving away, indicating the availability of open space to drive into, without having to fixate them directly.

This study demonstrates that the gaze of field hockey players can be measured in a dynamic setting using modern wearable eye trackers (and analysis methods). The utilisation of mobile eye trackers in an ecological setting can also be expanded to other sports, considering that measuring gaze behaviour is sport‐specific. However, the application of high frequency mobile eye trackers comes with the difficulty of handling very large sets of raw gaze data (Kredel et al. 2017). To facilitate the practical implementation of gaze behaviour analysis in dynamic training settings, coaches and trainers would benefit from efficient software programmes capable of quickly analysing the data generated by high‐frequency mobile eye trackers. Another recommendation for future research is to repeat our analyses for (larger groups of) hockey players with different competition levels to assess the generalisability of our findings. Additionally, in future studies it would be also interesting to measure the fixation duration in the analysis, another potentially important factor in determining the usefulness of relevant information (Oudejans et al. 2012; Kredel et al. 2017). In our study, we chose to focus more on the spatial locations of gaze allocation rather than the time they spent viewing those locations.

## 5 Conclusion

In conclusion, this study represents one of the first examinations of decision‐making and gaze behaviour in a dynamic field‐hockey scenario. The findings revealed differences in gaze behaviour when players made correct decisions compared to incorrect decisions in specific representative game scenarios. The requirement to hit a ball appears to alter gaze in important ways when compared to other invasive team sports such as basketball in which the ball is directly controlled by the ball carrier. In order to execute their decision, the final fixation in every hockey situation was towards the ball, showing that players prioritise information from the ball over other players or the space. However, rather than the final or second‐to‐last fixation, it was the first fixation that players made in each scenario which was most decisive of whether they made a correct decision or not. Scanning the open space instead of immediately fixating on a specific player appeared to be beneficial in decision‐making.

## Conflicts of Interest

The authors declare no conflicts of interest.
